# A Prospective Cohort Study on the Development of Claw Horn Disruption Lesions in Dairy Cattle; Furthering our Understanding of the Role of the Digital Cushion

**DOI:** 10.3389/fvets.2020.00440

**Published:** 2020-07-28

**Authors:** Bethany E. Griffiths, Philippa J. Mahen, Rachel Hall, Nikolaos Kakatsidis, Nick Britten, Kerry Long, Lara Robinson, Hannah Tatham, Rebecca Jenkin, Georgios Oikonomou

**Affiliations:** Department of Livestock and One Health, Institute of Infection, Veterinary & Ecological Sciences, University of Liverpool, Liverpool, United Kingdom

**Keywords:** dairy cattle, lameness, claw horn disruption lesion, digital cushion, mastitis

## Abstract

Claw horn disruption lesion (CHDL) is the collective term used to describe non-infectious foot lesions such as sole ulcers (SU), sole hemorrhage (SH), and white line disease (WLD) that commonly affect dairy cattle. The potential role of the bovine digital cushion, an anatomical structure located under the pedal bone and composed mostly of adipose and connective tissue, in the aetiopathogenesis of CHDL has recently been the subject of several studies. The aim of this prospective cohort study is to identify risk factors associated with the development of CHDL and to add further evidence regarding the role of the digital cushion. In order to achieve that we collected data from 500 lactations; 455 dairy cows from 3 farms were enrolled in this study. Data were collected from each animal on three occasions: 3–4 weeks before expected calving date, 1 week post calving, and 8–10 weeks post-calving. At each occasion, sole soft tissue thickness (the combined depth of the digital cushion and corium, SSTT) was measured using B-mode ultrasonography. At 8–10 weeks post-calving foot trimming was undertaken and the presence of CHDLs was recorded. Univariable analysis was undertaken between variables of interest, before multivariable regression models were constructed. Mixed effects multivariable linear regression models were created to describe the changes in SSTT and associations with various explanatory variables. Multivariable logistic regression models with the presence of SU, SH, or WLD as an outcome were also built. SSTT was shown to decrease from calving to early lactation (EL). Primiparous animals were found to have smaller SSTT, than multiparous animals. Animals with greater BCS had greater SSTT. Cows with a SU in early lactation had lower SSTT both at pre-calving and calving inspections comparing to cows without a SU. Cows that developed mastitis within 30 days of calving had approximately four times higher odds of developing SU compared to cows that did not develop mastitis. Our study advances our understanding of animal level risk factors associated with the development of CHDL and highlights the importance of the periparturient period.

## Introduction

Lameness is one of the greatest challenges facing the dairy industry, given the severe negative impacts on animal welfare (1), fertility (2), and milk yield (3). It is associated with substantial economic losses (4) and is still highly prevalent within dairy herds (5). Claw horn disruption lesion (CHDL) is the collective term used for non-infectious lameness causing lesions such as sole ulcers (SU), sole hemorrhage (SH), and white line disease (WLD) that commonly affect dairy cattle (6). Lesions which make up CHDL are thought to be different presentations of a similar disease process (7), with SH preceding SU (8–10). Although widely recognized as a significant issue within the dairy industry their aetiopathogenesis is yet to be fully elucidated. Current research suggests these lesions are the result of contusions of the corium under the third phalanx (11). The insult causes hemorrhage and necrosis of the keratinocytes within the sole corium, reducing the ability of the cow to synthesize new claw horn in affected feet and resulting in CHDLs (6, 8).

A cow's suspensory apparatus is rudimentary in comparison to horses (12). To compensate, cows have a better developed digital cushion (DC) which supports a significantly larger proportion of their body weight. This structure was first studied in 1999 by Kofler et al. (13), and described further by Räber et al. (12). A thinner DC has been associated with increased risk of developing CHDL (14, 15) and it has been hypothesized that the DC becomes thinner as cows mobilize body fat after calving (16). A recent study by Newsome et al. (17) showed that digital cushion thickness (DCT) decreased during the periparturient period but this thinning could not be entirely explained by fat mobilization alone.

Furthermore, the process of parturition (calving) has been associated with increased laxity in the connective tissue supporting the distal phalanx within the claw (18), quite possibly due to the hormonal effect of relaxin or estrogen. Systemically induced inflammation around calving may also compromise the suspensory apparatus via activating matrix metalloproteinases, which in turn play a central role in the degradation of its connective fibers. In addition, proinflammatory mediators associated with direct stimulation of lipolysis (19) could also lead to fat mobilization from the DC and a reduction of the latter's protective properties. We have previously shown that early signs of local inflammation were indeed associated with reduced DCT in the beginning of lactation and before the development of detectable CHDL (20).

Given the severe, wide ranging negative effects, coupled with the high prevalence of cows afflicted with CHDL, research is required to further elucidate their aetiopathogenesis; this could translate to better prevention strategies. Research has associated the depth of the DC with the development of CHDLs however most studies examined cows at a single time point. The main objective of this prospective cohort study is to add further evidence as to how the DCT changes over the peri-parturient period and its association with the development of CHDL. The importance of other animal level risk factors is also investigated.

## Methods

### Farm Recruitment and Ethics

The study was approved by the University of Liverpool Veterinary Research Ethics Committee (Reference VREC269). Data from 500 lactations were collected from 455 Holstein cows on three commercial dairy farms in the North-West of England and North Wales. The study was conducted between December 2014 and December 2015 on one of the three collaborating farms. The study was then continued on all three collaborating farms during the period between January 2017 and September 2017. The farms were selected due to their proximity to the Institute of Veterinary Science (University of Liverpool) and their willingness to collaborate with our research group.

### Farm Characteristics

On farm 1, the milking parlor and one third of the waiting area floor was rubber matting on concrete. All other walkways were grooved concrete. Cows were housed in cubicle sheds. Concrete based cubicles were lined with various mattress types (rubber, gel) and bedded with sawdust. Pen passageways were grooved concrete and were automatically scraped two to three times an hour. Dry cows were housed in sheds with a deep straw lying area and a grooved concrete loafing area. Youngstock were housed in cubicles during the winter months, and at pasture in the summer month.

On farm 2, the milking parlor was concreted and no matting was present in the parlor or the collecting yard. All other walkways were grooved concrete. Cows were housed in cubicle sheds. High yielding cows had access to concrete based cubicles with mats and shallow sand. Low yielding and freshly calved cows had access to deep sand bedded cubicles. Pen passageways were grooved concrete and automatically scraped two to three times an hour. Dry cows were housed on pasture during the summer and on deep sand bedded cubicles during winter. Youngstock were housed on concrete based cubicles with straw from weaning until first service.

On farm 3, matting was present in the parlor. All other walkways were grooved concrete, with matting present on the exit of the parlor and down the main race. Cows were housed in deep sand bedded cubicle sheds. Pen passageways were grooved concrete and scraped three times a day with a tractor. Dry cows were housed on a separate unit with deep sand bedded cubicles. Youngstock were housed on a separate unit with cubicles.

The diets for all the cows were formulated according to NRC guidelines. All cows were scheduled for routine foot trimming, at drying off and at ~60 days in milk for farm 1 and farm 3. Farm 2 cows were scheduled for routine foot trimming at drying off. Lame cows received additional foot trimming as necessary by farm staff. Footbaths, consisting of 4% copper sulfate twice weekly and 3% formalin once weekly, were located in the exit lane of the milking parlor at farm 1, whilst cows in Farm 2 were footbathed three times a week in 3% formalin on exit of the milking parlor. Farm 3 used a 3% formalin footbath once daily, located in the exit lane of the milking parlor.

### Data Collection

Data were collected from each animal on three occasions: 3–4 weeks from the expected calving date, 1 week post-calving and 8–10 weeks post-calving, referred to as pre-calving, fresh, and early lactation (EL), respectively. On each occasion mobility was assessed, using the AHDB 0–3 scale scoring method (21) by observing the cow walking on a flat surface. Body condition score (BCS) was assessed using the Penn State method; scores were between 1 and 5 in 0.25 increments (1= very thin, 5 = obese) (22).

Cows were restrained in a foot trimming crush for measurement of DCT using an Easi-Scan ultrasound machine (sonographic B-mode, IMV Imaging, Bellshill, UK) equipped with a linear probe 5–8 MHz set at 5 MHz. The ultrasound machine settings were kept unchanged throughout the study. All measurements of DCT were undertaken at the midline, on the lateral claw of the hind left foot. To measure the DCT the foot was cleaned and loose horn was removed with a hoof knife, as described by Kofler et al. (13). Sole contact with the transducer was made using ultrasound gel (Ultrasound Gel, Henry Schein) and a gel standoff (Flexi gel standoff, IMV Imaging, Bellshill, UK). After freezing the image on the ultrasound monitor (Easi-Scan Ultrasound Remote Display, IMV Imaging, Bellshill, UK), measurements were taken to the nearest millimeter. Digital cushion thickness (DCT) was measured just dorsally to the tuberculum flexorum of the pedal bone at the typical SU site. The distance from the inner margin of the sole (identified as a thin echogenic line) to the distal edge of the pedal bone (identified as a thick echogenic line) was assessed. The anatomical area of the DC targeted for ultrasonography was the middle pad (11). The DCT measured here, is better described as the sole soft tissue thickness (SSTT), as both the DC and corium are included within the measurement taken.

When data were collected at 8–10 weeks post-calving (EL), both hind feet were trimmed using the Dutch five step method (23) and any visible foot lesions were recorded before SSTT was measured. The recorded lameness causing foot lesions were digital dermatitis, SU, WLD, SH, and interdigital hyperplasia. Cases were defined following the ICAR Claw Health Atlas definitions (24).

All other factors included in analysis, including calving date, age in days, parity, and important health information such as incidence of retained fetal membranes, milk fever, ketosis, mastitis, metritis, endometritis, and displaced abomasum, were obtained from the farms' management software.

### Statistical Analysis

Data were analyzed using JMP Pro 14 (SAS Institute Inc., Cary, NC). Univariable analyses were undertaken between variables, before multivariable regression models were constructed. Parity was fitted in all models as a categorical variable with 3 levels (1 for animals in their first parity, 2 for animals in their second parity, and 3 for animals in their third or greater than third parity). SSTT was used as a continuous variable but was also turned into a categorical variable with 3 levels (3 terciles with 1 including the cows with the lowest SSTT measurements and 3 for those with the greatest SSTT measurements); this allowed for a more straightforward interpretation of logistic regression models outputs. Similarly, BCS was also turned into a categorical variable with 3 levels (level 1 for BCS <2.5, level 2 for BCS from 2.5 to 3, and level 3 for BCS >3).

In order to describe changes in SSTT and its association with CHDL a mixed effects multivariable linear regression model was used. The continuous dependent variable was SSTT and the following independent variables were originally offered to the model: Body condition score, calving season (Spring, Summer, Autumn, Winter), study (1 for data collected between December 2014 and December 2015, and 2 for data collected between January 2017 and September 2017), parity, time point of measurement (pre-calving, fresh and EL), assessor, and presence or absence of CHDL (SU, WLD, SH). These variables were offered to the model either because they were found to be associated with SSTT in univariable analyses (*P* ≤ 0.20) or because they were of particular interest for this study (CHDL). Cow id nested within farm was fitted in the model as a random effect to account for within animal clustering of SSTT measurements. The covariance structure used was that of compound symmetry. Associations between explanatory variables were also investigated to identify collinearity between variables. Interaction terms of interest that were offered to this model were: time point of measurement by presence or absence of CHDL. Variables and their interactions were removed from the model manually and in a stepwise manner (with the variable with the highest *P*-value removed at each step), and only variables with *P* < 0.10 (*F*-test) were kept in the final model. If an interaction term was found to be significant, then the main effects were kept in the final model whether they were significant or not. The restricted maximum likelihood approach was taken when fitting the model. Rows with missing data were not included in the analysis. When two variables were both found to be significant but also strongly associated to each other (this was the case for assessor and study, with three assessors only participating in Study 1 and one assessor only participating in Study 2) the variable that led to a higher adjusted *R*^2^ was kept in the final model. Residuals by model predicted values, studentized residuals, and residuals normal quantile plots were visualized in order to evaluate the model's goodness of fit and that assumptions of normality and homoscedasticity were met. Leverage plots (partial-regression residual leverage plots) for all fixed effects included in the model were also visualized. For categorical explanatory variables results are presented as least squares means ± standard error of the mean. Pairwise comparisons of least squares means were made using the Tukey-Kramer Honestly Significant Difference (HSD) test.

Logistic regression models with presence of SU, WLD, or SH at the EL inspection as an outcome were also built. Variables with a *P* ≤ 0.20 in the univariable analyses were offered to these multivariable logistic regression models. Variables were removed from the models manually and in a stepwise manner (with the variable with the highest *P*-value removed at each step), and only variables with *P* < 0.10 (likelihood ratio test) were kept in the final model. Explanatory variables originally offered in the model with SU as an outcome were: SSTT terciles at fresh and EL, calving season (Winter, Spring, Summer, and Autumn), study (1 for data collected between December 2014 and December 2015, and 2 for data collected between January 2017 and September 2017), incidence of clinical mastitis the first 30 days post calving, BCS group at EL, and parity. Explanatory variables originally offered in the model with SH as an outcome were: SSTT terciles at EL, calving season, study, and parity. Explanatory variables originally offered in the model with WLD as an outcome were: calving season, study, BCS group at pre-calving and EL, and parity. Farm was included in all the logistic regression models whether or not it was found to be significant. The Lack of Fit test was used to evaluate models goodness of fit and the likelihood ratio test was used to determine the overall significance of the models. The predictive ability of each one of the final three logistic regression models was assessed with receiver operating characteristic analysis and the calculated area under the curve. Results from logistic regression models are presented as Odds Ratios. *P*-values and 95% confidence intervals (CI) for calculated Odds Ratios are Wald based estimates. All comparisons between different levels of categorical explanatory variables are for the odds of developing CHDL (SU, SH, WLD) vs. the odds of not developing CHDL.

## Results

Four hundred and fifty-five cows were enrolled, 45 of them for two consecutive lactations, totaling 500 lactation enrolments. Of the 1,500 ultrasound assessments due, 137 were missed [50 at pre-calving, 42 at fresh and 45 at early lactation (EL)]. Reasons for missing assessments were failure of sorting gate, termination of measurements due to animal stress presenting a risk for animal or researcher safety, removal from herd, and injury preventing foot trimming. Five assessors carried out the ultrasound assessments (assessor 1; *n* = 517 assessments, assessor 2; *n* = 519, assessor 3; *n* = 57, assessor 4; *n* = 94, assessor 5; *n* = 170; unrecorded; *n* = 6). The median time of assessment relative to calving was −17 days for pre-calving, 6 days for fresh and 67 days for EL. Table 1 shows summary data for the study population and assessments. More calvings occurred in spring than other seasons (spring *n* = 266, summer *n* =99, autumn *n* =53, winter *n* =82). There were 144 parity one animals, 134 parity two, and 222 parity three and over. Median BCS of the population decreased from pre-calving to fresh to EL (pre-calving 3.25, fresh 3, EL 2.5). Twenty animals had a case of mastitis in the first 30 days of lactation. From the 462 foot trims/examinations carried out at the EL time point, 52 cases of sole ulcer (SU) were found, 105 cases of sole hemorrhage (SH) and 80 cases of white line disease (WLD). Descriptive statistics results are presented in Table 1.

**Table 1:** Summary data for study populations.

Enrolled lactations (cows)	Farm 1	312 (267)
	Farm 2	75 (75)
	Farm 3	113 (113)
	Total	500 (455)
Missing ultrasound assessments	Pre-calving	50
	Fresh	42
	Early lactation	45
Total ultrasound assessments	1,363	
Time of assessments relative to calving (days) Median (range)	Pre-calving	−17 (−131–0)
	Fresh	6 (0–12)
	Early lactation	67 (37–97)
Season at calving	Spring: 266	
	Summer: 99	
	Autumn: 53	
	Winter: 82	
Parity	1: 144	
	2: 134	
	≥3: 222	
Study*parity	Study 1	Parity 1: 54
		Parity 2: 44
		Parity ≥3: 86
	Study 2	Parity 1: 90
		Parity 2: 90
		Parity ≥3: 136
Body condition score median (range)	Pre-calving	3.25 (2.25–4.5)
	Fresh	3 (1.75–4)
	Early lactation	2.5 (1.5–3.5)
Mastitis in first 30 days of lactation (primary cases)	20	
Claw horn disruption lesions (*n* = 462)	Sole ulcer: 52	
	Sole hemorrhage: 105	
	White line disease: 80	
Number of assessments per assessor	1: 517	
	2: 519	
	3: 57	
	4: 94	
	5: 170	

Results obtained from Univariable analyses with SSTT and presence of SU, SH, or WLD as outcome variables are presented in Supplementary Tables 1–4.

Results obtained from multivariable mixed effects linear regression analysis with SSTT as an outcome are presented in Table 2. Time point (*P* = 0.003), parity (*P* < 0.0001), BCS (*P* = 0.031), time point by SU interaction (*P* = 0.036), and assessor (*P* < 0.0001) were statistically significantly associated with SSTT. The statistically significant time point by SU interaction highlighted that changes of SSTT across time points were different between animals that developed a SU in EL and the ones that did not. There was no significant difference between SSTT at pre-calving and fresh in cows that did not develop SU (adjusted means 8.73 ± 0.14 and 8.65 ± 0.12 mm, respectively), whereas SSTT at EL was statistically significantly lower than the other two time points (8.27 ± 0.13 mm). In cows with SU, SSTT was at its lowest immediately after calving (however this was only a numeric difference that was not statistically significant). Parity one animals had significantly thinner SSTT than parity two or three and over animals (8.55 ±0.11, 9.30 ± 0.11, and 9.54 ± 0.10 mm, respectively). Animals with higher BCS had higher SSTT, with an estimated increase in SSTT of 0.3 mm for every one point increase in BCS. Model's adjusted *R*^2^ was 0.35.

**Table 2:** Results from multivariable mixed effects linear regression model for outcome sole soft tissue thickness (SSTT) (mm).

**Explanatory**	**Category**	**Adjusted**	**Standard**	**Tukey's**	***P*-value**
**variable**		**mean**	**error**	**HSD**	
Parity	1	8.55	0.11	A	<0.0001
	2	9.30	0.11	B	
	≥3	9.54	0.10	B	
Time point*sole ulcer	Pre-calving, no SU	8.73	0.14	A	0.036
	Pre-calving, SU	8.54	0.27	ABC	
	Fresh, no SU	8.65	0.12	AB	
	Fresh, SU	8.02	0.25	BC	
	Early lactation, no SU	8.27	0.13	C	
	Early lactation, SU	8.45	0.25	ABC	
Assessor	1	7.81	0.11	C	
	2	8.39	0.12	B	
	3	8.97	0.23	AB	<0.0001
	4	9.17	0.19	A	
	5	8.40	0.15	B	
BCS	Continuous variable	Estimate 0.30	0.14		0.031
Random effect		Variance Component			Percentage of total variance
CowID (nested within farm)		0.43	0.09		16.26
Residual		2.22	0.10		83.74

*HSD, honestly significant difference. Levels within a variable with different letters are statistically significantly different (P < 0.05)*.

Results obtained from multivariable logistic regression analysis with SU, SH, or WLD as an outcome are presented in Table 3. The multivariable logistic regression model for outcome SU retained BCS at EL, mastitis in the first 30 days of lactation, SSTT tercile at fresh and parity, along with the forced variable farm. The odds for having a SU were higher for animals with a BCS of <2.5 at EL than those with a BCS of 2.5–3.0 (OR 3.59, 95% CI 1.78–7.26, *P* = 0.0004). Animals that had a case of mastitis in the first 30 days of lactation displayed higher odds of having a SU than those that did not get mastitis (OR 3.97, 95% CI 1.31–12.09, *P* = 0.015). Animals with a SSTT at fresh of 8–9.5 mm and those with SSTT at fresh <8 mm had higher odds of developing a SU than those in with SSTT >9.5 mm (OR 2.20, 95% CI 1.02–4.73, *P* = 0.044, and 2.40, 95% CI 0.92–6.23, *P* = 0.074, respectively). The numeric difference between animals with SSTT at fresh <8 mm and those with SSTT>9.5 mm was marginally not statistically significant. Animals in their second parity had lower odds of getting SU than animals in their third or greater than third parity (OR 0.39, 95% CI 0.16–0.97 *P* = 0.043). This model's AUC was 0.77.

**Table 3:** Results from multivariable logistic regression models for outcomes SU, SH, and WLD.

**Outcome**	**Explanatory variable**	**Level**	**OR**	**95% CI**	***P*-value**
Sole Ulcer	BCS category at early lactation	<2.5	3.59	1.78–7.26	0.0004
		>3.0	0.58	0.07–4.65	0.60
		2.5–3.0		Reference	
	Mastitis in first 30 days of lactation	Yes	3.97	1.31–12.09	0.015
		No		Reference	
	SSTT tercile at fresh	<8 mm	2.40	0.92–6.23	0.074
		8–9.5 mm	2.20	1.02–4.73	0.044
		>9.5 mm		Reference	
	Parity	1	0.88	0.38–2.05	0.77
		2	0.39	0.16–0.97	0.043
		3		Reference	
		1	1.79	0.73–4.34	0.20
	Farm	2	2.53	0.86–7.41	0.09
		3		Reference	
Sole hemorrhage	Parity	3	0.56	0.32–0.97	0.0006
		2	0.30	0.15–0.59	0.039
		1		Reference	
	SSTT tercile at early lactation	<8 mm	1.86	1.05–3.29	0.034
		8–9.5 mm	2.21	1.21–4.04	0.010
		>9.5 mm		Reference	
	Season	Winter	1.42	0.69–2.92	0.35
		Summer	2.52	1.29–4.93	0.007
		Autumn	1.12	0.48–2.64	0.78
		Spring		Reference	
		1	3.76	1.53–9.22	0.004
	Farm	2	3.20	1.20–8.54	0.02
		3		Reference	
White line disease	Parity	3	7.68	3.16–18.66	<0.0001
		2	4.44	1.72–11.47	0.002
		1		Reference	
		1	4.00	1.75–9.19	0.001
	Farm	2	1.48	0.50–4.37	0.47
		3		Reference	

*Presented Odds Ratios (OR) are for each level against the reference category for the odds of developing SU, SH, or WLD; P-values and 95% confidence intervals (CI) are Wald based estimates*.

In the model with SH as an outcome variable (Table 3), parity, SSTT tercile at EL, season, and farm remained as significant. Animals in their second parity and animals in their third or greater than third parity had lower odds of developing SH comparing to primiparous animals (OR 0.30, 95% CI 0.15–0.59, *P* = 0.039; OR 0.56, 95% CI 0.32–0.97, *P* = 0.0006). Animals with a SSTT in the bottom (<8 mm) or middle tercile (8–9.5 mm) at EL had higher odds of having SH than those in the top tercile (>9.5 mm) (OR 1.86, 95% CI 1.05–3.29, *P* = 0.034; OR 2.21, 95% CI 1.21–4.04, *P* = 0.010, respectively). Animals that calved in summer had higher odds of developing SH than those calved in spring. This model's AUC was 0.73.

Only parity and farm were retained as significant in the model for WLD. Parity two and parity three or greater than three animals had higher odds of developing WLD than those in parity one (OR 4.44, 95% CI 1.72–11.47, *P* = 0.002; OR 7.68, 95% CI 3.16–18.66, *P* < 0.0001, respectively). This model's AUC was 0.72.

## Discussion

We measured SSTT at three time points from pre-calving to EL and found it to be at its thinnest in EL, in line with previously published work (16). Primiparous animals were found to have thinner SSTT compared to multiparous animals. Cows displaying a SU in EL were shown to have numerically thinner SSTT both at pre-calving and calving than those without SU. Body condition was shown to be positively correlated with SSTT. Cows that developed mastitis within the first 30 days of calving had almost four times higher odds of developing a SU in EL comparing to cows that did not develop mastitis. Variables found to have an effect on the development of SU, SH, and WLD included parity, with parity two and parity three or greater animals showing lower odds of developing SH, whilst showed higher odds of developing WLD. Those animals with a SSTT in the middle tercile around calving were at higher odds of developing SU compared to those in the top tercile, whilst animals with a SSTT in the bottom or middle tercile at EL were at higher odds of developing a SH than those in the top tercile.

SSTT in our study was observed to be at its thinnest in EL. This is similar to previously published results (16), where SSTT was at its lowest from 30 to 120 days post-calving; however this study was cross sectional and unable to provide strong evidence regarding the change in SSTT over a lactation. This nadir at EL could be due to cows experiencing a negative energy balance, resulting in partial fat mobilization from the SST (12), as suggested by Bicalho et al. (16). A recent prospective cohort study by Newsome et al. (17) observed the thinnest point to be around calving, with an increase in thickness observed from calving to EL. The authors suggested that this could be due to increased laxity of the suspensory apparatus associated with calving, causing the distal phalanx to compress the SST.

The inconsistency regarding when in lactation the SSTT nadir occurs between our study and the Newsome et al. (17) study could be due to the difference in the fat pad targeted. A recent short communication by Hiss-Pesch et al. (25) reported that the fat pads within the digital cushion are not uniform and that fat could be mobilized from them at different rates. It could also be due to different farm management systems or differences in genetics. The farms used by Newsome et al. (17) featured automated milking systems, whereas all three farms within the current study used conventional herringbone or rotary milking parlors. This will affect the time cows spend on their feet and could potentially be associated with post-calving changes in SSTT (26). Work undertaken by Oikonomou et al. (27) described a heritability estimate of 0.33 for SSTT, therefore genetics may also play a role in the inconsistences presented by these two studies. Contrary to the Newsome et al. (17) study where the assessor measuring SSTT was blinded to stage of lactation, we assessed SSTT knowing whether the cows were at pre-calving, fresh, or EL stage. Therefore, unconscious bias in our measurements cannot be precluded and is another possible explanation for the observed discrepancy between the two studies. This is further discussed in the “study limitations” section of our discussion.

Our results show that cows within the middle tercile for SST thickness immediately after calving had approximately four times higher odds of developing SU than cows in the upper tercile. This finding was similar to that presented in 2009 by Bicalho et al. (16). Toholj et al. (14) also showed that cows with a SSTT below 3 mm had four times higher odds of developing a SU than cows with a SSTT above 3 mm. Furthermore, cows within both of the lower terciles for SSTT at EL were at greater odds of developing SH compared to those cows in the upper tercile, a finding supported by Newsome et al. (28). Our findings support the hypothesis that the time around calving is important in the development of CHDL's.

Cows that developed a SU in EL had lower SSTT during the pre-calving period than cows which did not develop SU and experienced a greater thinning of the SST around parturition. Newsome et al. (17) was able to show that cows which develop a SU or severe SH had thinner SSTT, yet thinning of the SST was not significantly associated with the development of CHDLs. Bicalho et al. (16) showed that cows with lesions, regardless of parity, had significantly thinner SSTT, whilst thin SSTT were associated with cows that had CHDLs in the same lactation, and cows that go on to develop CHDL's in the subsequent lactation (15). Previous work revealed that cows affected by lameness and CHDLs undergo new bone growth at the plantar and palmar aspect of the distal phalanx (29). These exostoses may reduce the DC capacity to protect cells within the corium from being contused resulting in further inflammation, and further development of exostoses and CHDLs. Another possible explanation for our findings is that the relaxation of the suspensory apparatus described by Tarlton et al. (18) to occur around calving period could be exacerbated in cows developing SU; the reasons behind this remain unknown but could be associated with the animals' genetic make-up. Several regions within the genome have recently been identified as being significantly associated with SSTT at calving (30).

Body condition score and SSTT were significantly associated, in agreement with previous studies (15–17). In EL, when body condition was at its lowest, SSTT was also at its thinnest. An increase of one condition score was associated with an increase in SSTT of 0.3 mm. This represents a smaller magnitude of effect than the results presented by Bicalho et al. (16) however is larger than those results presented by Newsome et al. (18). We also found that cows with a BCS of <2.5 at EL had higher odds of having SU in EL compared to cows with a BCS of 2.5 to 3. It has been shown by multiple studies that low BCS is associated with the development of lameness but can also a result of it (31, 32); cows with a low BCS at parturition had 9.4 times higher odds of developing lameness throughout lactation compared with better conditioned cows (33).

Parity was shown to be significantly associated with both the SSTT and the odds of developing SU, SH, and WLD. Primiparous cows were found to have significantly thinner SST compared to multiparous cows, which is supported by the existing literature (12, 16, 17). Our results have shown that primiparous animals had higher odds of developing SH compared to multiparous animals, but had lower odds of developing WLD. This finding was again supported by previous findings (16, 34). One hypothesis is that the composition of the developing digital cushion has a somewhat protective function (35), together with the reduced forces going through the foot of comparatively lighter animals (12, 36) around calving when the insult is expected to have occurred, especially in primiparous animals where calving is expected to be more challenging as they are added to the herd and undergo parturition for the first time. Additionally, given these are naïve animals which are unlikely to have experienced CHDL's, exostosis is unlikely to significantly affect the development of these lesions. Therefore, SH occurs rather than a SU or WLD, given SH is thought to be a precursor or result from a milder insult (10). Animals in their third or greater than third parity were at higher odds of developing SU comparing to animals in their second parity. The effect of exostosis, digital cushion composition, increased force through feet and the stress of calving could increase the risk of animals in their third or greater than third parity forming SU and WLD over SH. However, animals in their third or greater than third parity did not have a significantly higher SU incidence than primiparous animals and this suggests a “non-linear” association between parity and incidence of SU, with second parity animals having the lowest incidence. This contradicts previous findings that suggested that SU incidence is lower in primiparous animals (37). The reason behind this finding is unclear; a possible explanation is that animals in their second parity may benefit from a more developed DC (comparing to primiparous animals) but are yet to experience the increased risk associated with multiple calvings and chronic inflammation that may be more evident in animals in their third or greater than third parity.

Cows that developed mastitis within 30 days of calving had almost four times higher odds of developing SU compared to those cows that did not develop mastitis. Clinical mastitis in the early lactation period has been linked with lameness (38), however not with CHDLs specifically. This highlights the potential role of early lactation systemic inflammation in the development of CHDL. The effect of systemic inflammation on the suspensory apparatus has been hypothesized to lead to CHDL. However, our study cannot clearly show such cause and effect relationships. Another likely explanation of our findings could be that cows with mastitis spend longer periods of time standing because of the discomfort associated with the disease and this predisposes them to the development of SU. An unknown common link associated with the aetiopathogenesis of both early lactation mastitis and SU development is another plausible explanation of our findings.

Our study has limitations that need to be taken into consideration when interpreting our findings. Multiple assessors were used for the measurement of SSTT. This has been accounted for in our model with SSTT as an outcome but not in the models where SSTT was an explanatory variable. When assessing SSTT, the assessor was not blinded to the stage of lactation and although no conscious bias was present the possibility of unconscious bias cannot be precluded. We have used a different dataset (collected as part of a larger, ongoing study) including repeated SSTT measurements on 136 cows in order to further investigate this (data not shown). These measurements were taken by the same assessor who was blinded to stage of lactation (or any other relevant information). Analysis of this dataset confirms our findings regarding the associations between parity and SSTT, and the association between SSTT at calving and presence of CHDL in EL. On the other hand, in this analysis, SSTT is not at its lowest at EL, but immediately after calving [similarly to the study by Newsome et al. (17)]. This would indeed suggest that an element of unconscious bias in the presented here study cannot be precluded. A larger scale study with measurements taken by the same, blinded, assessor would potentially help in clarifying this issue. Claw horn disruption lesion information was analyzed by animal in this study and not by claw, as undertaken by Newsome et al. (17), and no distinction was made between animals displaying these lesions on the studied claw and animals displaying these lesions on a different claw. Given inflammation is suggested to play an important role in the SSTT of cows affected with CHDLs, an important improvement in this study would be to include CHDL information by claw. Finally, another limitation of our study has to do with the fact that we only measured SSTT on lifted feet (similarly to the majority of studies on SSTT). Bach et al. (39) recently showed that measurements of SSTT on weight bearing feet yielded different results to the measurements taken on lifted feet. This study was only conducted on 10 animals so must be interpreted with caution but does however suggest that had we been able to measure SSTT on weight bearing feet our results could have been different.

## Conclusion

This prospective cohort study found that SSTT significantly decreased from calving to EL and that SSTT at calving was associated with the development of SH and SU. The results presented are in general in line with some of the previously published literature. Parity was found to be significantly associated both SSTT and the development of SH, SU, and WLD. We have also shown an association between early lactation mastitis and the development of SU.

## Data Availability Statement

The raw data supporting the conclusions of this article will be made available by the authors, without undue reservation.

## Ethics Statement

The animal study was reviewed and approved by University of Liverpool Veterinary Research Ethics. Written informed consent was obtained from the owners for the participation of their animals in this study.

## Author Contributions

Data collection was undertaken by BG, NK, NB, LR, HT, RJ, RH, KL, and GO. Statistical analysis was undertaken by GO and assisted by PM. The manuscript draft was written by BG with significant contributions from PM and GO. GO conceived and designed the study and provided with funding. All authors have approved the final version of this manuscript and agree to be accountable for all aspects of the work. All authors contributed to the article and approved the submitted version.

## Conflict of Interest

The authors declare that the research was conducted in the absence of any commercial or financial relationships that could be construed as a potential conflict of interest.
