# Supplementary material for: A Prospective Cohort Study on the Development of Claw Horn Disruption Lesions in Dairy Cattle; Furthering our Understanding of the Role of the Digital Cushion
Source: Front Vet Sci. 2020 Jul 28;7:440. doi: 10.3389/fvets.2020.00440 (PMC7399069; doi:10.3389/fvets.2020.00440)
Supplement: Supplementary file 1 [file Table_1.docx]

Supplementary Table 1. Results from univariable analyses for outcome sole soft tissue thickness (SSTT) (mm). Linear regression analysis was used for continuous variables, whilst categorical explanatory variables underwent analysis of variance.

| **Explanatory variable** | **Level** | **Mean** | **Standard error** | **P value** |
| --- | --- | --- | --- | --- |
| Study | One | 9.36 | 0.07 | <.0001 |
|  | Two | 8.56 | 0.06 |  |
| Time | Pre-calving | 9.07 | 0.08 | <.0001 |
|  | Fresh | 8.96 | 0.08 |  |
|  | Early Lactation | 8.57 | 0.08 |  |
| Parity | 1 | 8.27 | 0.08 | <.0001 |
|  | 2 | 8.95 | 0.09 |  |
|  | ≥3 | 9.21 | 0.07 |  |
| Assessor | 1 | 8.48 | 0.07 | <.0001 |
|  | 2 | 8.90 | 0.07 |  |
|  | 3 | 9.67 | 0.22 |  |
|  | 4 | 9.88 | 0.17 |  |
|  | 5 | 9.05 | 0.13 |  |
| Season | Spring | 8.74 | 0.06 | 0.0469 |
|  | Summer | 9.04 | 0.11 |  |
|  | Autumn | 9.01 | 0.14 |  |
|  | Winter | 8.96 | 0.12 |  |
| Presence of SU | 0 | 8.88 | 0.05 | 0.4865 |
|  | 1 | 8.77 | 0.14 |  |
| Presence of WLD | 0 | 8.79 | 0.05 | 0.003 |
|  | 1 | 9.14 | 0.10 |  |
| Presence of SH | 0 | 8.89 | 0.05 | 0.338 |
|  | 1 | 8.78 | 0.10 |  |
| Body condition score |  | **Estimate**  0.18 | 0.10 | 0.0887 |
