# Supplementary material for: A Prospective Cohort Study on the Development of Claw Horn Disruption Lesions in Dairy Cattle; Furthering our Understanding of the Role of the Digital Cushion
Source: Front Vet Sci. 2020 Jul 28;7:440. doi: 10.3389/fvets.2020.00440 (PMC7399069; doi:10.3389/fvets.2020.00440)
Supplement: Supplementary file 2 [file Table_2.docx]

Supplementary Table 2. Results from univariable contingency table analyses with presence of a sole ulcer (SU) at early lactation as an outcome. Sole soft tissue thickness (SSTT) is grouped into terciles. Likelihood ration testing was used to obtain P values.

| **Explanatory variable** | **Category** | **Prevalence of cows with a SU at early lactation** | **P value** |
| --- | --- | --- | --- |
| Farm | 1 | 11.38% | 0.2289 |
|  | 2 | 16.18% |  |
|  | 3 | 7.69% |  |
| Parity | 1 | 9.85% | 0.0121 |
|  | 2 | 5.56% |  |
|  | ≥3 | 15.69% |  |
| Season | Spring | 10.29% | 0.0221 |
|  | Summer | 9.78% |  |
|  | Autumn | 4.00% |  |
|  | Winter | 20.78% |  |
| Mastitis within 30 days of calving | No | 10.36% | 0.01 |
|  | Yes | 33.33% |  |
| SSTT at pre-calving | 1 | 12.16% | 0.526 |
|  | 2 | 11.29% |  |
|  | 3 | 8.18% |  |
| SSTT at fresh | 1 | 13.41% | 0.1865 |
|  | 2 | 13.64% |  |
|  | 3 | 7.84% |  |
| SSTT at early lactation | 1 | 13.97% | 0.1444 |
|  | 2 | 6.48% |  |
|  | 3 | 11.85% |  |
| BCS at pre-calving | <2.5 | 0.00% | 0.6608 |
|  | 2.5 to 3 | 12.36% |  |
|  | >3 | 10.00% |  |
| BCS at fresh | <2.5 | 0.00% | 0.6174 |
|  | 2.5 to 3 | 12.32% |  |
|  | >3 | 9.59% |  |
| BCS at early lactation | <2.5 | 21.09% | 0.0005 |
|  | 2.5 to 3 | 7.49% |  |
|  | >3 | 8.70% |  |

BCS= Body Condition Score
