# Supplementary material for: A Prospective Cohort Study on the Development of Claw Horn Disruption Lesions in Dairy Cattle; Furthering our Understanding of the Role of the Digital Cushion
Source: Front Vet Sci. 2020 Jul 28;7:440. doi: 10.3389/fvets.2020.00440 (PMC7399069; doi:10.3389/fvets.2020.00440)
Supplement: Supplementary file 3 [file Table_3.docx]

Supplementary Table 3. Results from univariable contingency table analyses with presence of a sole haemorrhage (SH) at early lactation as an outcome. Sole soft tissue thickness (SSTT) is grouped into terciles. Likelihood ration testing was used to obtain P values.

| **Explanatory variable** | **Category** | **Prevalence of cows with SH at early lactation** | **P value** |
| --- | --- | --- | --- |
| Farm | 1 | 28.62% | <.0001 |
|  | 2 | 20.59% |  |
|  | 3 | 7.69% |  |
| Study | 1 | 30.17% | 0.0026 |
|  | 2 | 18.02% |  |
| Parity | 1 | 33.33% | 0.0003 |
|  | 2 | 12.70% |  |
|  | ≥3 | 22.06% |  |
| Season | Spring | 14.81% | <.0001 |
|  | Summer | 40.22% |  |
|  | Autumn | 24.00% |  |
|  | Winter | 25.97% |  |
| Mastitis within 30 days of calving | No | 22.52% | 0.6102 |
|  | Yes | 27.78% |  |
| SSTT at pre-calving | 1 | 22.97% | 0.9845 |
|  | 2 | 22.04% |  |
|  | 3 | 22.01% |  |
| SSTT at fresh | 1 | 25.61% | 0.8871 |
|  | 2 | 23.23% |  |
|  | 3 | 22.88% |  |
| SSTT at early lactation | 1 | 29.41% | 0.0054 |
|  | 2 | 27.78% |  |
|  | 3 | 16.11% |  |
| BCS at pre-calving | <2.5 | 0.00% | 0.549 |
|  | 2.5 to 3 | 24.16% |  |
|  | >3 | 20.77% |  |
| BCS at fresh | <2.5 | 0.00% |  |
|  | 2.5 to 3 | 20.29% | 0.201 |
|  | >3 | 27.40% |  |
| BCS at early lactation | <2.5 | 22.66% |  |
|  | 2.5 to 3 | 22.80% | 0.549 |
|  | >3 | 26.09% |  |

BCS= Body Condition Score
