# Supplementary material for: A Prospective Cohort Study on the Development of Claw Horn Disruption Lesions in Dairy Cattle; Furthering our Understanding of the Role of the Digital Cushion
Source: Front Vet Sci. 2020 Jul 28;7:440. doi: 10.3389/fvets.2020.00440 (PMC7399069; doi:10.3389/fvets.2020.00440)
Supplement: Supplementary file 4 [file Table_4.docx]

Supplementary Table 4. Results from univariable contingency table analyses with presence of a white line disease (WLD) at early lactation as an outcome. Sole soft tissue thickness (SSTT) is grouped into terciles. Likelihood ratio testing was used to obtain P values.

| **Explanatory variable** | **Category** | **Prevalence of cows with WLD at early lactation** | **P value** |
| --- | --- | --- | --- |
| Farm | 1 | 22.41% | 0.0002 |
|  | 2 | 11.76% |  |
|  | 3 | 6.73% |  |
| Study | 1 | 24.02% | 0.0028 |
|  | 2 | 13.07% |  |
| Parity | 1 | 4.55% | <.0001 |
|  | 2 | 17.46% |  |
|  | ≥3 | 25.49% |  |
| Season | Spring | 14.40% | 0.1024 |
|  | Summer | 22.83% |  |
|  | Autumn | 26.00% |  |
|  | Winter | 14.29% |  |
| Mastitis within 30 days of calving | No | 17.34% | 0.9405 |
|  | Yes | 16.67% |  |
| SSTT at pre-calving | 1 | 16.22% | 0.4085 |
|  | 2 | 16.13% |  |
|  | 3 | 21.38% |  |
| SSTT at fresh | 1 | 12.20% | 0.2335 |
|  | 2 | 17.68% |  |
|  | 3 | 20.92% |  |
| SSTT at early lactation | 1 | 14.71% | 0.4731 |
|  | 2 | 15.74% |  |
|  | 3 | 19.43% |  |
| BCS at pre-calving | <2.5 | 0.00% | 0.1147 |
|  | 2.5 to 3 | 22.47% |  |
|  | >3 | 15.00% |  |
| BCS at fresh | <2.5 | 0.00% | 0.5545 |
|  | 2.5 to 3 | 19.20% |  |
|  | >3 | 15.75% |  |
| BCS at early lactation | <2.5 | 19.53% | 0.13 |
|  | 2.5 to 3 | 17.59% |  |
|  | >3 | 4.35% |  |

BCS= Body Condition Score
